# Association between the General Practitioner Workforce Crisis and Premature Mortality in Hungary: Cross-Sectional Evaluation of Health Insurance Data from 2006 to 2014

**DOI:** 10.3390/ijerph15071388

**Published:** 2018-07-02

**Authors:** János Sándor, Anita Pálinkás, Ferenc Vincze, Valéria Sipos, Nóra Kovács, Tibor Jenei, Zsófia Falusi, László Pál, László Kőrösi, Magor Papp, Róza Ádány

**Affiliations:** 1 Department of Preventive Medicine, Faculty of Public Health, University of Debrecen, 4028 Debrecen, Hungary; palinkas.anita@sph.unideb.hu (A.P.); vincze.ferenc@sph.unideb.hu (F.V.); sipos.valeria@sph.unideb.hu (V.S.); kovacs.nora@sph.unideb.hu (N.K.); jenei.tibor@sph.unideb.hu (T.J.); adany.roza@sph.unideb.hu (R.Á.); 2 Department of Financing, National Health Insurance Fund, 1139 Budapest, Hungary; falusi.zs@neak.gov.hu (Z.F.); pal.l@neak.gov.hu (L.P.); korosi.l@neak.gov.hu (L.K.); 3 National Institute for Health Development, Budapest, Diószegi St 64, 1113 Budapest, Hungary; papp.magor@nefi.hu; 4 MTA-DE-Public Health Research Group, University of Debrecen, 4028 Debrecen, Hungary; 5 WHO Collaborating Centre on Vulnerability and Health, Department of Preventive Medicine, Faculty of Public Health, University of Debrecen, 4028 Debrecen, Hungary

**Keywords:** primary health care, workforce crisis, general practitioner vacancy, aging of general practitioners, premature mortality

## Abstract

The workforce crisis of primary care is reflected in the increasing number of general medical practices (GMP) with vacant general practitioner (GP) positions, and the GPs’ ageing. Our study aimed to describe the association between this crisis and premature mortality. Age-sex-standardized mortality for 18–64 years old adults was calculated for all Hungarian GMPs annually in the period from 2006 to 2014. The relationship of premature mortality with GPs’ age and vacant GP positions was evaluated by standardized linear regression controlled for list size, urbanization, geographical location, clients’ education, and type of the GMP. The clients’ education was the strongest protective factor (beta = −0175; *p* < 0.001), followed by urban residence (beta = −0.149; *p* < 0.001), and bigger list size (beta_1601–2000_ = −0.054; *p* < 0.001; beta_2001−X_ = −0.096; *p* < 0.001). The geographical localization also significantly influenced the risk. Although GMPs with a GP aged older than 65 years (beta = 0; *p* = 0.995) did not affect the risk, GP vacancy was associated with higher risk (beta = 0.010; *p* = 0.033), although the corresponding number of attributable cases was 23.54 over 9 years. The vacant GP position is associated with a significant but hardly detectable increased risk of premature mortality without considerable public health importance. Nevertheless, employment of GPs aged more than 65 does not impose premature mortality risk elevation.

## 1. Introduction

A fundamental attribute of the health care sector is extraordinary labor intensity. The effectiveness in this sector is highly dependent upon health care staff quantity and quality. A general trend that has been observed due to the recently manifested shortage of health professionals in almost all developed countries is an increasing demand for health care staff that is steadily being met to declining degrees. In this respect, the evolving workforce crisis has become a major problem in many developed countries. It has also been well documented that primary health care (PHC) is among the most affected areas of the health sector [1,2,3].

Almost all Member States of the European Union face critical workforce shortages. Although reliable data on the health care labor market are sometimes not available, it has been forecasted that shortages in the number of health professionals will amount to approximately 970,000 (230,000 physicians; 590,000 nurses; and 150,000 dentists, pharmacists, and physiotherapists) by 2020. Further, because the new recruits cannot replace the retirees, the age of working physicians has been and probably will continue to be steadily increasing [4]. Especially, missing and ageing physicians impose a current challenge to health policy in the PHC setting, because the ratio of general practitioners to specialists has also been decreasing in most developed countries [5].

With regards to the workforce crisis, Hungary is a typical European country, where the supply of general practitioners (GPs) steadily decreased between 2002 and 2006, and 48% of GPs were aged above 55 years. According to the Primary Health Care Activity Monitor for Europe project, the Hungarian primary care workforce development index was in the midrange of countries within Europe [6,7,8].

European Union initiatives to mitigate the workforce crisis have been restricted to the improvement of workforce planning methodologies, support of health professionals teaching program development, facilitation of the sharing of countries’ experiences in workforce recruitment and retention strategies, and support of the WHO Global Code on the international recruitment of health personnel implementation [4,9]. Although the importance of these initiatives cannot be disputed, it seems that the problems in PHC cannot be handled without thorough restructuring. In other words, instead of focusing the main efforts to recruitment of more physicians with improved knowledge and skills (to continue an approach that has been unsuccessful at increasing GP supply), the structure of PHC should be modernized, adapted to current needs determined by the increasing burden of chronic diseases in ageing societies, new technologies that are available in PHC, and shift from hospital care to the provision of care closer to home [10,11,12,13].

Although there has been some consensus on the necessity of implementing innovative solutions and fundamental reforms, these policy decisions have been postponed [14]. Although there are data on the consequences of structural weaknesses in PHC, it seems that the lack of convincing data on the health losses resulting from the PHC workforce crisis hinders the decision-making process. Presumably, more data on the impact of the workforce crisis (missing and ageing GPs) may help facilitate the implementation of innovative solutions in the restructuring of PHC.

Because PHC varies widely between different countries [15], the manifestations and consequences of a variable workforce crisis may also vary widely country by country. Unfortunately, in Hungary and in countries of the former Soviet Bloc [16,17,18], the consequences of this crisis have not yet been investigated, neither in regard to the processes of care provided nor the health status of the population receiving care. Even the very basic health status indicator of premature mortality has not yet been evaluated [19].

Our study aimed to evaluate the role of GP age and vacancy on premature mortality risk among adults after adjustment for basic GMP structural factors.

## 2. Materials and Methods

The study included all GMPs in Hungary and evaluated the period from 2006 to 2014. The number of deaths of any cause in the target population was counted for each year and GMP. The study population for each year and GMP was defined as adults 18–64 years old who had not changed GMP in the 5 years prior to the investigated year. The data for this analysis was provided by the National Health Insurance Fund (NHIF).

Annual and GMP-specific indirect standardized mortality ratios (SMRs) were computed. National reference mortality rates were calculated for each year by gender and age groups (as 18–19, 20–24, 25–29, 30–34, 35–39, 40–44, 45–49, 50–54, 55–59, 60–64 years). Annual and GMP-specific expected number of deaths were determined using national stratum-specific reference rates and by the demographic composition of the GMPs. Ratio of observed and expected numbers of deaths were computed to obtain SMRs.

GMPs were characterized by year. GMPs providing care for adults only and for adults and children were categorized. GMPs were categorized according to the number of adult patients for which care was provided (less than 800, 801–1200, 1201–1600, 1601–2000, and 2001 or more patients). GMPs were also categorized as having vacant or filled GP position (provided by temporary GP with availability restricted in time and place, or by permanent contracted GP with continuous availability) and being rural or urban. GP age was classified as 65 years or younger, and older than 65 (at least 66) years. The geographical location of each GMP was described by its county.

The socioeconomic status of adults receiving care at each GMP was approximated by gender- and age-standardized relative education (rEDU). Using 2011 Hungarian Census data (provided by the Hungarian Central Statistical Office), years of education were calculated for the Hungarian population by gender and age groups (as 18–19, 20–24, 25–29, 30–34, 35–39, 40–44, 45–49, 50–54, 55–59, 60–64 years). The expected number of school years was determined for adults in each settlement and year by demographic characteristics of the settlement and national reference values of 2011. The ratio of observed and expected values indicated the rEDU of people living in a certain settlement. For GMPs providing care for adults living in one settlement, settlement-specific rEDU was considered as a GMP parameter. For GMPs providing care for adults from more than one settlement, the weighted settlement-specific rEDU was calculated, where the weights were defined by the distribution of clients’ places of living. The dichotomized rEDU was used in linear regression analysis. The median rEDU was the applied threshold.

Aggregated proportions of rural practices, GMPs providing care for adults and children, average rEDU and practice size were calculated for vacant practices, for practices with GPs age 65 years or younger, and for practices with GPs older than 65 years. These aggregated measures were evaluated by their corresponding 95% confidence intervals.

Multiple linear regression models were used to analyze the associations between year-specific GMP parameters and SMRs normalized by Box-Cox transformation [20]. Linear regression coefficients (b) were computed, and standardized linear regression coefficients (beta) were also calculated to ensure the direct comparability of effect size of risk factors. *p*-values calculated for explanatory parameters were considered as significant if they were less than 0.05. Semipartial correlation coefficients were computed to estimate the impact of explanatory factors on premature mortality. Statistical analysis was performed by PASW Statistics 18.

The administrative database of NHIF used for the analysis did not contain client-specific data. The protocol for this aggregate data investigation was reviewed in the respect of ethical and legal requirements and approved by the Internal Data Safety and Patient Rights Board of the NHIF (OEP: E01/317-1/2014). This board is responsible for preventing violation of clients’ right for data security, according to the Act on Protection of Health and Health Related Personal Data (47/1997).

## 3. Results

### 3.1. GMP Characteristics

The number of investigated GMPs and their client population for each year varied between 4759 and 4813 and between 5,979,558 and 6,028,690, respectively. The sums of observed GMP-years and client-years were 43,111 and 53,780,309, respectively. Altogether, 576,344 (1.06%) out of 54,356,653 client-years were not included in the analysis because of the GMP-changing within 5 years. The majority of GMPs were urban (this proportion increased slightly from 62.43% to 66.26% during the study period), and provided care only for adults (consistently approximately 69%). The distribution of GMP size did not change significantly over the study period, apart from the significant increase of the proportion of GMPs served less than 800 patients. Due to internal indirect standardization, the mean rEDU was 1, with a standard deviation of 0.16. (Table 1)

**Figure 1:**
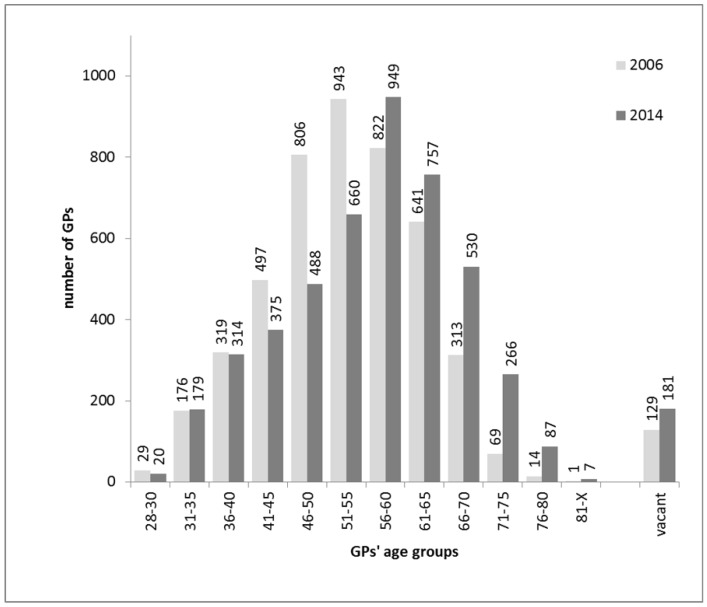
Changes in the age distribution of Hungarian general practitioners responsible for the provision of primary health care to adults and number of vacant practices between 2006 and 2014.

**Figure 2:**
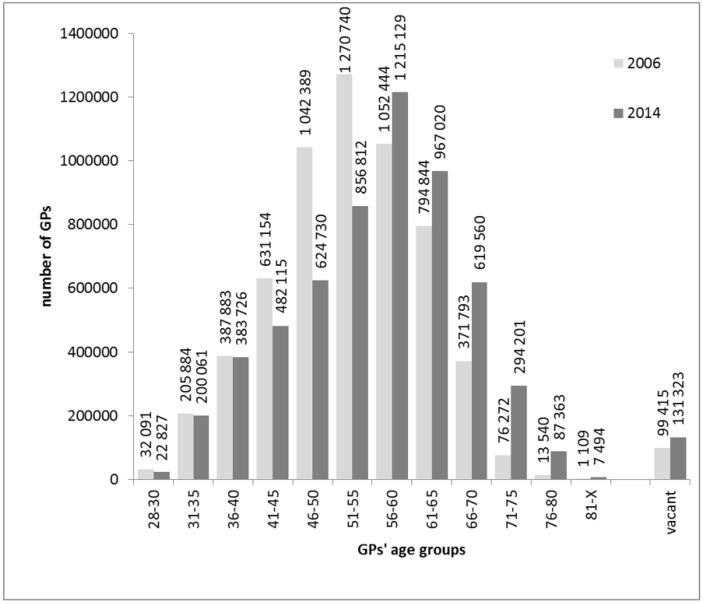
Number of 18–64-year-old adults receiving care from general medical practices by general practitioner (GP) age and in practices with vacant GP post between 2006 and 2014 in Hungary.

The increase in the proportion of GPs aged at least 66 years (from 10.19% to 21.25%) was profound. There was also a remarkable increase in the proportion of GMPs with vacancies (from 2.71% to 3.76%) (Figure 1). The number of patients aged 18–64 years who received care from GPs above 65 years increased significantly (from 462,714 to 1,008,618 patients). There were 99 415 and 131 323 adults aged less than 65 in 2006 and 2014 receiving care in vacant GMPs, respectively (Figure 2).

Throughout the entire study period, 246,285 deaths were registered. Annual and GMP-specific SMRs ranged from 0 to 2.72. The average (±SE) annual and GMP-specific SMRs was 1.030 (±0.003).

### 3.2. GMP Characteristics by Vacancy Status and Age of GP

Practices that were rural, provided care for adults and children, and of small (less than 1200 list) size were overrepresented among GMPs with vacant GP posts. The less than median rEDU was significantly more frequent among adults who received care in vacant practices.

GPs above 65 were overrepresented in urban GMPs and in GMPs providing care for adults only. Higher patient rEDU was associated with higher than 65 years of GP age. GPs less than 66 years were underrepresented in small practices. (Table 2).

### 3.3. Risk Factors for Premature Death

The aggregated SMR for GMPs with vacant GP position was significantly elevated in the whole investigated period (SMR = 1.25; *p* < 0.001). The similar measure for GMPs provided by GPs aged more than 65 did not deviate from the Hungarian reference (SMR = 0.99; *p* = 0.101).

The Box-Cox transformed GMP-specific SMRs were normally distributed by Kolmogorov-Smirnov test (*p* = 0.200). According to the multivariate linear regression analysis, the strongest protective factor against premature death was higher level of education (beta = −0.175; *p* < 0.001). Urban residence was also associated with a lower risk of premature death (beta = −0.149; *p* < 0.001). Receiving care in bigger GMPs reduced the mortality risk (beta_1601–2000_ = −0.054; *p* < 0.001; beta_2001−X_ = −0.096; *p* < 0.001). A significantly higher risk of premature death was associated with GMPs that provided care for adults only (beta = 0.039; *p* < 0.001) compared to GMPs providing care for adults and children.

Six counties (Borsod-Abaúj-Zemplén, Szabolcs-Szatmár-Bereg, Komárom-Esztergom, Jász-Nagykun-Szolnok, Nógrád, Pest) were associated with a higher risk than the reference county (Budapest, the capital of Hungary). The risk of premature mortality was significantly lower in another five counties (Zala, Veszprém, Csongrád, Tolna, Győr-Moson-Sopron).

Receiving care in a GMP with vacant GP post (beta = 0.010; *p* = 0.033) was, while that with a GP aged 66 years or older (beta = 0.0003; *p* = 0.995) was not, associated with higher risk of premature death than receiving care from a GMP with a GP aged less than 65 years. The number of premature deaths attributable to GP vacancy during the period from 2006 to 2014 was 23.54. (Table 3).

## 4. Discussion

### 4.1. Main Findings

The strong risk factor nature of the vacant GP position for premature death described by the age- and sex-standardized mortality ratios was not confirmed by the multivariate analysis. That is, most of the considerable risk elevation suggested by unadjusted analysis could be explained by the structural attributes of GMPs. Although the GP vacancy proved to be a statistically significant weak risk factor, it is of negligible public health importance for premature death.

On the other hand, the other manifestation of the PHC workforce crisis, the higher than 65 year of GPs, proved to be a risk factor in neither the univariate nor in the multivariate analyses.

### 4.2. Other Findings

Our investigation found that GMP-specific premature mortality risk among adults who had not changed GMP in the 5 years before the analysis varied widely, and this variability depended mainly on GMP characteristics such as urban residence, list size of GMP, geographical position of the county where the GMP is located, and rEDU approximated socio-economic status of population cared for by the GMP. These factors were evaluated as important risk factors both by regression analysis (indicated by at least 5% risk elevation for involved practice according to the standardized regression coefficients) and by assessing attributable cases (indicated by at least 50 excess cases per year—that is, 450 excess cases in the 9 investigated years—in the whole country according to the number of attributable deaths). There was a good agreement in ranking risk factors according to their importance for a practice and for the whole primary care.

### 4.3. Observations in International Context

These findings are in harmony with previous findings on the association between GMP level premature mortality and socioeconomic status [21,22,23] and urban setting [24].

Receiving care in a GMP of smaller size seems to be not a protective factor in Hungarian settings. This observation deviates from the published experiences on the protective effect of better GP supply and shorter list size [22,23]. The likely explanation is that the Hungarian clients have free choice to be registered in a GMP. The selection of GP is influenced by the trust of clients, which is affected by their perceived effectiveness of care. The higher the clients’ trust, the bigger the list size can be reflected in this observation.

The significant county-associated risks (reflecting the specialty of health care, life style, and environmental safety not controlled by the level of education in our analysis) varied remarkably (from beta = −0.026 to beta = 0.074). This finding verified the public health importance of geographical position of GMP, in concordance with the well-known spatial variability of premature death within countries [25,26,27].

Our results demonstrated that temporary GPs providing care in a vacant GMP have restricted effectiveness in managing patients compared to the effectiveness achieved by a permanent GP in Hungary, are in concordance with the demonstrated risky nature of the lack of continuity in PHC [22,23,28,29,30].

Although there are scarce publications on the negative association between high physicians’ age and patient outcomes [31,32], according to our observations, the experience of GPs of retirement aged (above 65 years) could counterbalance their possibly limited capacities in PHC organization.

### 4.4. Strengths and Limitations

Because our investigation covered the entire country, the results describe the association between GMP characteristics and premature mortality among adults without selection bias. The high number of cases ensured high statistical power, enabling the identification of both strong and weak influencing factors.

Both GMP characteristics and premature mortality were quantified based on NHIF-GMP contracts and death certification. Therefore, misclassification of outcome or explanatory variables was avoided.

The only explanatory parameter that was not available for every investigated year was education, potentially resulting in some misclassification in the study. However, because education status remains unchanged over certain age in adults, this misclassification may not have had a strong influence.

This analysis was restricted to patients who did not change their GMP in the 5 years before the evaluation to avoid bias associated with patient migration.

The major limitation of this study was that the GMPs, instead of patients, were the unit of analysis [33]. This restriction was a consequence of NHIF’s data availability, which has to respect the clients’ right for data protection.

Further, the 2011 Hungarian Census did not collect detailed data on socio-economic status (e.g., income, occupation) but for the level of education. Therefore, the education could be applied as proxy indicator of socio-economic status.

Unfortunately, there are no available indicators for Hungarian GMPs which describe the disease burden among GMPs’ clients. Consequently, an additional limitation of the study was the missing control for the morbidity status of clients. Although this factor is correlated with and consequently was controlled partly by education, the mortality risk caused by existing disease was not taken into consideration.

Because clients in Hungary can choose and change the GP without any legal restriction, it is likely that patients who need thorough care of a GP are more willing to change GP than a patient/client without this need. Due to this movement, the risk of premature death is also shifted towards the GMPs with permanent GPs. This resulted in the underestimation of the risk elevation of premature death in GMPs with vacant GP positions. Because of the lack of morbidity monitoring in NHIF the size of this underestimation could not be assessed.

### 4.5. Further Research Needs

Our results demonstrate that the GMP-level workforce crisis manifested in vacant GP positions leads to a statistically significant increased risk of premature death among adults without significant public health importance on the level of the countrywide effectiveness of PHC. It seems to be probable that the weak premature mortality risk elevation is associated with more pronounced elevation of morbidity and life quality impairment related risks. The identification of the mechanisms behind this risk association (that is, the identification of which components of the GPs’ activity are sensitive to providing service in a practice with vacant GP post, and the identification of patient related factors which increase their sensitivity for consequences of GP vacancy) needs further investigation to find interventions through which temporary GPs should be supported.

### 4.6. Implications

Taking into consideration the negligible population-level consequences of GP post vacancy, the development of innovative PHC structural interventions (facilitated by many other unsolved PHC problems as well), which are not predominantly targeted towards increasing of the number of physicians employed in PHC but on collaboration with non-medical health professionals (physiotherapists, dieticians, psychologists, public health experts, etc.) at the PHC level through the delegation of certain activities under the supervision of GPs [34,35,36], are not urged by premature mortality data, at present. However, the proportion of vacant GMPs has shown steady increase. Therefore, the importance of the workforce crisis as a risk factor will most likely increase. This potential impact can be an additional facilitating factor for shifting interventions from the recently applied recruitment enforcement to deeper structural reform of the primary care, in the long term.

## 5. Conclusions

Although the tested model did not include some important risk factors that limits the validity of our observations, the workforce crisis in PHC seems to be weakly associated with increased risk of premature mortality among adults in Hungary. At the level of a GMP exposed to GP vacancy, the risk elevation is hardly detectable, and the impact of GP vacancy on the performance of PHC in the whole country is negligible, at present. However, considering the increasing proportion of GMPs with vacant GP posts, the public health importance of this workforce crisis as a risk factor will most likely be increased. On the other hand, this study showed that the facilitation of labor market interventions to improve job opportunities for older GPs can help to overcome temporarily problems associated with vacant GMPs without introducing new premature mortality risk factor into PHC.

## Figures and Tables

**Table 1:** Distribution of general medical practice (GMP) characteristics by year in Hungary during 2006–2014 according to the National Health Insurance Fund registration.

GMP Characteristics	2006	2007	2008	2009	2010	2011	2012	2013	2014	2006–2014
**Providing service for adults and children (%)**	1475	1498	1480	1473	1476	1480	1487	1486	1481	13,336
	(30.99)	(31.41)	(30.95)	(30.77)	(30.80)	(30.83)	(30.93)	(30.96)	(30.77)	(30.93)
**Providing service for adults only (%)**	3284	3271	3302	3314	3316	3321	3321	3314	3332	29,775
	(69.01)	(68.59)	(69.05)	(69.23)	(69.20)	(69.17)	(69.07)	(69.04)	(69.23)	(69.07)
**Rural (%)**	1788	1802	1797	1796	1784	1665	1667	1624	1624	15,547
	(37.57)	(37.79)	(37.58)	(37.52)	(37.23)	(34.68)	(34.67)	(33.83)	(33.74)	(36.06)
**Urban (%)**	2971	2967	2985	2991	3008	3136	3141	3176	3189	27,564
	(62.43)	(62.21)	(62.42)	(62.48)	(62.77)	(65.32)	(65.33)	(66.17)	(66.26)	(63.94)
**Age of GP (%)**										
**Vacant (%)**	129	149	127	121	127	135	149	166	181	1284
	(2.71)	(3.12)	(2.66)	(2.53)	(2.65)	(2.81)	(3.10)	(3.46)	(3.76)	(2.98)
**X-65 years**	4145	4067	4050	3990	3921	3870	3923	3694	3609	35,269
	(87.10)	(85.28)	(84.69)	(83.35)	(81.82)	(80.61)	(81.59)	(76.96)	(74.98)	(81.81)
**66-X years**	485	553	605	676	744	796	736	940	1023	6558
	(10.19)	(11.60)	(12.65)	(14.12)	(15.53)	(16.58)	(15.31)	(19.58)	(21.25)	(15.21)
**Size of GMP (%)**										
**X-800 clients**	83	108	101	102	110	127	401	141	157	1330
	(1.74)	(2.26)	(2.11)	(2.13)	(2.30)	(2.65)	(2,80)	(2.94)	(3.26)	(3.09)
**801–1200 clients**	609	609	595	601	638	655	1523	663	670	6563
	(12.80)	(12.77)	(12.44)	(12.55)	(13.31)	(13.64)	(13,73)	(13.81)	(13.92)	(15.22)
**1201–1600 clients**	1531	1500	1492	1484	1480	1477	1797	1495	1526	13,782
	(32.17)	(31.45)	(31.20)	(31.00)	(30.88)	(30.76)	(30,96)	(31.15)	(31.71)	(31.97)
**1601–2000 clients**	1542	1580	1604	1600	1576	1555	810	1530	1508	13,305
	(32.40)	(33.13)	(33.54)	(33.42)	(32.89)	(32.39)	(32,14)	(31.88)	(31.33)	(30.86)
**2001-X clients**	994	972	990	1000	988	987	277	971	952	8131
	(20.89)	(20.38)	(20.70)	(20.89)	(20.62)	(20.56)	(20,40)	(20.23)	(19.78)	(18.86)
**Standardized education (%)**										
**Less than median level**	2373	2408	2384	2384	2387	2392	2394	2390	2386	21,498
	(49.86)	(50.49)	(49.85)	(49.80)	(49.81)	(49.82)	(49.79)	(49.79)	(49.57)	(49.87)
**Above median level**	2386	2361	2398	2403	2405	2409	2414	2410	2427	21,613
	(50.14)	(49.51)	(50.15)	(50.20)	(50.19)	(50.18)	(50.21)	(50.21)	(50.43)	(50.13)
**Number of premature deaths**	29,282	29,553	28,525	28,485	27,450	26,792	26,279	25,135	24,784	246,285
**Number of registered clients**	5,979,558	5,988,278	6,019,392	6,028,690	5,987,701	5,976,905	5,976,288	5,931,136	5,892,361	53,780,309
**Total number of GMPs**	4759	4769	4782	4787	4792	4801	4808	4800	4813	43,111

**Table 2:** Distribution of structural attributes and standardized mortality ratios of general medical practices according to the lack of permanent GP and to the age of GP in Hungary during 2006–2014 (with 95% confidence intervals).

	GP Age X-65 Years	GP Age 66 ≤ Years	Vacant	Hungary
Proportion of practices provided care for adults only	69.46% [68.98–69.94]	73.24% [72.17–74.31]	36.84% [34.2–39.48]	69.07% [68.63–69.5]
Proportion of urban practices	64.39% [63.89–64.88]	69.49% [68.37–70.6]	23.29% [20.97–25.6]	63.94% [63.48–64.39]
Proportion of practices with less than 800 list size	1.86% [1.72–2.00]	4.18% [3.69–4.66]	31.15% [28.62–33.69]	3.09% [2.92–3.25]
Proportion of practices with 801–1200 list size	13.63% [13.27–13.98]	19.91% [18.95–20.88]	35.12% [32.51–37.74]	15.22% [14.88–15.56]
Proportion of practices with 1201–1600 list size	31.76% [31.28–32.25]	34.78% [33.63–35.93]	23.21% [20.9–25.52]	31.97% [31.53–32.41]
Proportion of practices with 1601–2000 list size	32.41% [31.92–32.9]	26.94% [25.87–28.02]	8.33% [6.82–9.85]	30.86% [30.43–31.3]
Proportion of practices with more than 2000 list size	50.11% [49.59–50.63]	56.25% [55.05–57.45]	19.47% [17.3–21.64]	50.13% [49.66–50.61]
Above median standardized relative education of clients	50.11% [49.59–50.63]	56.25% [55.05–57.45]	19.47% [17.3–21.64]	50.13% [49.66–50.61]
Standardized mortality ratio	0.996 [0.992–1.001]	0.992 [0.981–1.002]	1.247 [1.215–1.280]	1 [0.996–1.004]

**Table 3:** Multivariate linear regression analysis of the associations between structural general medical practice (GMP) indicators and age- and gender-standardized annual and GMP-specific mortality ratios among adults aged 18–65 years who did not change GMP in the 5 years prior to the investigated year in Hungary during 2006–2014.

GMP Indicators	linear Regression Coefficient	*p*-Value	Standardized Linear Regression Coefficient	Semipartial Correlation Coefficient	Number of Attributable Cases
Type of GMP					
GMP for adults only/GMP for adults and children	**0.024**	**<0.001**	**0.039**	**0.025**	**154.37**
urban/rural	**−0.089**	**<0.001**	**−0.149**	**−0.100**	**−2441.45**
Age/vacancy of GP					
66-X years GP age/X-65 years GP age	0	0.995	0	0	0
vacant GMP/X-65 years GP ages	**0.018**	**0.033**	**0.010**	**0.010**	**23.54**
Size of GMP					
X-800 GMP size/1201–1600 GMP size	−0.009	0.279	−0.005	−0.005	−6.09
801–1200 GMP size/1201–1600 GMP size	0.008	0.058	0.010	0.009	18.69
1601–2000 GMP size/1201–1600 GMP size	**−0.033**	**<0.001**	**−0.054**	**−0.045**	**−509.24**
2001-X GMP size/1201–1600 GMP size	**−0.071**	**<0.001**	**−0.096**	**−0.082**	**−1647.91**
Relative education					
above median/less than median	**−0.101**	**<0.001**	**−0.175**	**−0.132**	**−4316.52**
Counties:					
Bács-Kiskun county/Budapest	−0.013	0.062	−0.010	−0.009	−18.12
Baranya county/Budapest	−0.008	0.276	−0.006	−0.005	−6.17
Békés county/Budapest	0.011	0.147	0.008	0.007	10.94
Borsod-Abaúj-Zemplén county/Budapest	**0.080**	**<0.001**	**0.074**	**0.061**	**909.57**
Csongrád county/Budapest	**−0.034**	**<0.001**	**−0.023**	**−0.021**	**−108.18**
Fejér county/Budapest	0.003	0.715	0.002	0.002	0.69
Győr-Moson-Sopron county/Budapest	**−0.029**	**<0.001**	**−0.02**	**−0.018**	**−81.13**
Hajdú-Bihar county/Budapest	0.002	0.735	0.002	0.002	0.60
Heves county/Budapest	−0.003	0.759	−0.002	−0.001	−0.49
Jász-Nagykun-Szolnok county/Budapest	**0.02**	**0.012**	**0.013**	**0.012**	**33.10**
Komárom-Esztergom county/Budapest	**0.023**	**0.008**	**0.013**	**0.012**	**36.43**
Nógrád county/Budapest	**0.025**	**0.008**	**0.013**	**0.012**	**36.32**
Pest county/Budapest	**0.012**	**0.036**	**0.012**	**0.010**	**22.83**
Somogy county/Budapest	0.010	0.188	0.007	0.006	9.00
Szabolcs-Szatmár-Bereg county/Budapest	**0.022**	**0.002**	**0.017**	**0.014**	**51.20**
Tolna county/Budapest	**−0.038**	**<0.001**	**−0.021**	**−0.019**	**−88.55**
Vas county/Budapest	−0.017	0.056	−0.010	−0.009	−18.94
Veszprém county/Budapest	**−0.041**	**<0.001**	**−0.026**	**−0.023**	**−135.19**
Zala county/Budapest	**−0.043**	**<0.001**	**−0.025**	**−0.023**	**−131.9**

Significant results with *p* < 0.05 in bold.

## Abbreviations

GP	general practitioner
GMP	general medical practice
rEDU	gender and age standardized relative education
PHC	primary health care
NHIF	National Health Insurance Fund
SMR	Standardized Mortality Ratio
